# Another traveler’s tale: African tick-bite fever

**DOI:** 10.1128/asmcr.00010-24

**Published:** 2025-01-07

**Authors:** A. Brunet, A. Leplatois, Clement Lier, A. Limelette, Y. N’Guyen

**Affiliations:** 1 Unité Post Urgences Médicales, Hôpital Robert Debré, CHU Reims, Reims, France; 2 Laboratoire de Virologie, Pôle de Biologie Territoriale, Reims, France; 3 Laboratoire de Bactériologie, Pôle de Biologie Territoriale, Reims, France; 4 UMR-S 1320 CardioVir, Université de Reims Champagne Ardenne, Reims, France; Pattern Bioscience, Austin, Texas, USA

**Keywords:** *Rickettsia africae*, eschar, tick, serology, PCR

## Abstract

**Background:**

In the present report, we discuss the clinical and species-specific diagnosis of African tick-bite fever.

**Case Summary:**

A 58-year-old man was admitted to the hospital for fever, headache, and myalgia. The clinical examination yielded only two small purpuric lesions with a dark center on the thigh and the abdomen. The patient reported that he had just returned from a 10-day trip to the eastern region of South Africa, and that he had hiked through the bush in Eswatini 7 days before the onset of fever. The white blood cell count showed lymphopenia, and the C reactive protein and alanine aminotransferase levels were mildly elevated. The diagnoses of bacteremia, malaria, COVID 19, and arboviruses were ruled out. Both the diagnoses of African tick-bite fever and Mediterranean spotted fever were plausible because both *Rickettsia africae* and *Rickettsia conorii* are present in South Africa. The presence of two skin lesions presumed to be necrotic eschars was a bit more suggestive of African tick-bite fever. Treatment with doxycycline was started once blood serology and a biopsy of the abdominal lesion had been performed. The outcome was rapidly favorable. A PCR assay performed on the abdominal eschar confirmed the involvement of *R. africae,* while the successive serological assays did not.

**Conclusion:**

Numerous necrotic eschars without secondary maculopapular rash affecting the sole and palms are more commonly observed in African tick-bite fever than in Mediterranean spotted fever.

## INTRODUCTION

The potential severity and mortality linked to rickettsial diseases have led physicians worldwide to keep a high degree of suspicion toward them (1, 2). Appropriate antibiotic therapy should be prescribed as soon as possible (3), once the species-specific clinical signs usually present have been sought (4). The diagnosis is subsequently routinely confirmed by serological assays, although a species-specific diagnosis is sometimes challenging (5). In the present case report, we discuss the clinical and species-specific diagnosis of African tick-bite fever.

## CASE PRESENTATION

**Fig 1:**
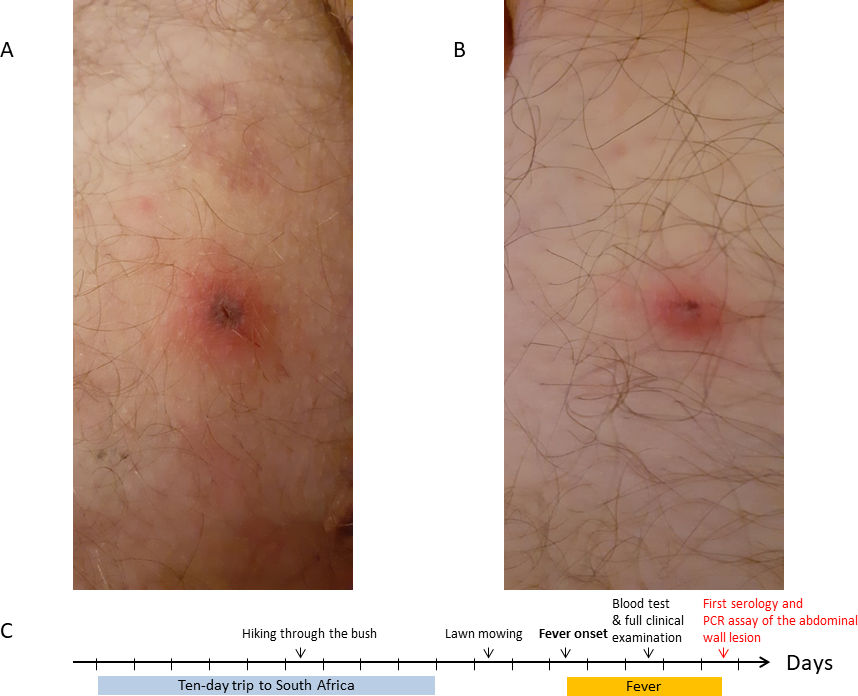
(**A**) Photograph of the right thigh (fourth day of fever). (**B**) Photograph of the abdomen (fourth day of fever). (**C**) Timeline of the present case.

The patient was a 58-year-old man who was admitted to the hospital for fever reaching 41°C for 3 days. He reported no significant past medical history, only headache and myalgia without stiffness of the neck. A full clinical examination only found right inguinal lymphadenitis with two small purpuric lesions with a dark center on the thigh and the abdomen (Fig. 1A and B). The white blood cell count showed only lymphopenia (800/mm^3^; normal range: 1000–4000/mm^3^). The C reactive protein (CRP) level (27 mg/L; normal value: under 10 mg/L) was mildly elevated, as was the alanine aminotransferase level (55 U/L; normal range: 7–40 U/L). Creatinine (84 µmol/L; normal range: 53–97 µmol/L) and aspartate aminotransferase (33 U/L, normal range 13–40 U/L) levels were within normal range. The electrocardiogram was normal.

The patient reported that he mowed his lawn at home (north-eastern France) 2 days before the onset of fever, and also that he had returned from a 10-day trip to the eastern region of South Africa with his wife 3 days before the onset of fever (Fig. 1C). A thorough questioning revealed that they both hiked through the bush in Mlilwane WildLife Sanctuary (Eswatini, formerly Swaziland) 7 days before the onset of fever (Fig. 1C). His wife, who was asymptomatic, used mosquito repellent creams or sprays for clothing during this hiking tour. The patient did not, but he did not report any tick or mosquito bites.

Blood cultures were negative at 5 days, while thick and thin blood smears did not demonstrate the presence of blood parasites, such as *Plasmodium* spp. The COVID 19 antigen test and the arbovirus (Chikungunya, Dengue, West Nile, Zika) PCR and serological assays were negative.

Some of the ward physicians considered that the diagnosis of African tick-bite fever was more or less confirmed, given the two skin lesions presumed to be necrotic eschars (also called “taches noires” or Pieris’ black spots) and the recent trip to South Africa. Others believed that the diagnoses of African tick-bite fever and Mediterranean spotted fever were both plausible. The first opinion was supported by the presence of several necrotic eschars corresponding to the primary sites of attachment of the infected ticks (6) belonging to the *Amblyomma* genus, which have an aggressive behavior (5). The existence of more than one necrotic lesion has previously been considered as highly suggestive of African tick-bite fever because it is observed for more than 50% of patients with African tick-bite fever (5). The second opinion was supported by the existence of both *Rickettsia africae* and *Rickettsia conorii* in South Africa (6, 7).

By mutual agreement, treatment with oral doxycycline, which is considered as the treatment of choice for all Rickettsial infections, regardless of the species involved, was started at a dose of 100 mg twice a day for 7 days once blood serology and biopsy of the abdominal lesion had been performed. The outcome was rapidly favorable: the fever went down, and the lymphocytes were within normal range after the first dose of doxycycline. The CRP level was 4 mg/L (normal value under 10 mg/L), and the patient was asymptomatic 4 days after the end of the 7-day course of doxycycline. A PCR assay was performed on the frozen biopsy of the abdominal eschar in order to identify the *Rickettsia* species involved with the help of local microbiologists and the National Reference Centre (Fig. 1C). The diagnosis of African tick-bite fever was confirmed here by the PCR assays. The successive routinely performed serological assays confirmed the diagnosis of Rickettsial infection without being able to determine the species involved (Table 1).

**TABLE 1:** Serological and PCR assays performed in the patient^*a*^

Assays performed	Samples taken on the fifth day of fever	Samples taken 14 days after the fifth day of fever
Commercially available chemiluminescent serology kit (Virclia, Vircell Granada, Spain) used on blood samples	IgG anti*-Rickettsia conorii*: negative	**IgG anti-*Rickettsia conorii*: positive**
	IgM anti*-Rickettsia conorii*: negative	**IgM anti*-Rickettsia conorii*: positive**
National Reference Centre indirect immunofluorescence assays performed on blood samples	Ig anti*-Rickettsia conorii*: negative	Ig anti*-Rickettsia conorii*: total Ig titer 50 (significant titer IgG > 128; IgM > 64)
	Ig anti*-Rickettsia typhi*: negative	Ig anti*-Rickettsia typhi*: negative
	Ig anti*-Rickettsia felis*: negative	Ig anti*-Rickettsia felis*: negative
National Reference Centre real-time PCR assay performed on eschar biopsy	**Spotted fever group: positive**	Not performed
	***Rickettsia africae*: positive**	Not performed

^*a*^ PCR, polymerase chain reaction; Ig, immunoglobulin. The significant positive results are written in bold.

## DISCUSSION

As reported in previous reviews (5, 7), *R. africae*, which is the species involved in the development of African tick-bite fever, is a Gram-negative intracellular bacterium belonging to the spotted fever group among the *Rickettsiaceae*. Except for *Rickettsia felis*, which is transmitted by fleas in the same way as *Rickettsia typhi* in the typhus group, all species in the spotted fever group are usually transmitted by infected ticks (5, 7). The species of the tick vector explains both the regionally endemic nature of some Rickettsial species (e.g., *Rickettsia rickettsii*, involved in Rocky Mountain spotted fever, only present in America [7]) and the clinical features of the necrotic eschars. The high infection rate and the aggressive behavior of the ticks belonging to the *Amblyomma* genus (the vector of *R. africae*) explain the more numerous necrotic eschars (5) than with *Rhipicephalus* ticks, the vector of *R. conorii* involved in the development of Mediterranean spotted fever, where there is usually just one necrotic eschar (6) (Table 2).

**TABLE 2:** Similarities and differences between some *Rickettsia* species infections

Species	*Rickettsia rickettsii*	*Rickettsia conorii*	*Rickettsia africae*	*Rickettsia slovaca*
Tick vector	*Dermacentor, Amblyomma, Rhipicephalus*	*Rhipicephalus*	*Amblyomma*	*Dermacentor*
Disease(s)	Rocky Mountain spotted fever and Brazilian spotted fever	Mediterranean spotted fever	African tick-bite fever	Scalp eschar and neck lymphadenopathy (SENLAT), formerly tick-borne lymphadenitis
Distribution	North, Central, and South America	Europe, North Africa, Sub-Saharan Africa, Middle East, Russia, and Asia	Sub-Saharan Africa, Central America, and Oceania	Europe, Middle East, Russia, and Asia
Clinical pattern				
Lymphadenitis	Usually absent in 70% of cases	Regional near the eschar	Regional near the eschar	Always cervical or occipital
Eschar	Almost always absent (8)	Almost always single in 98% of cases (2)	Multiple in more than 50% of cases	Single on the scalp
Rash from the third day of fever and onwards	Usually present: erythematous spots observed in 85–90% of cases, beginning on wrists and involving palms and soles in 20% of cases (9); purpuric eruption and gangrene in case of severe disease	Usually present: erythematous spots observed in 85–95% of cases and involving palms and soles in 70–75% of cases (2, 10)	Often absent: erythematous spots observed in 15 to 45% of cases (5)	Usually absent: erythematous spots observed in 0.2% of cases (11); one report of facial cellulitis (12)
Severity^*a*^	++	+	+/−	−

^*a*^ Increasing severity was graded semi-quantitatively between − and ++.

Increased vascular permeability and the disruption of endothelial cell junctions before the development of vasculitis and cytokine release are considered to be the main pathological processes in Rickettsial infections (5–7). Interestingly, African tick-bite fever, which is considered as a mild self-limited disease (5, 6), is less frequently associated with a diffuse secondary rash (maculopapular and affecting soles and palms from the third day onwards) than Rocky Mountain or Mediterranean spotted fever (5, 6). The hematological (lymphopenia) and biochemical parameter variations (CRP and aminotransferase elevation) are less marked in African tick-bite fever than in Mediterranean spotted fever (13).

In clinical practice, the rapid resolution of symptoms once a narrow-spectrum treatment active against all Rickettsial species, such as doxycycline, had been started can be suggestive of the diagnosis of Rickettsial infections, especially in the case of unexplained high fever with lymphopenia and mild CRP or aminotransferase elevation. Conversely, another diagnosis should be sought if the symptoms do not resolve with doxycycline, which is considered to be the most effective treatment by South African microbiologists (6). As other Rickettsial species, *R.africae* strains are reported to be universally susceptible to tetracyclines, as well as new macrolides or fluoroquinolones (5), although cultures and antimicrobial susceptibility testing can only be performed on the yolk sacs of developing chicken embryos or cell cultures (5) in a BioSafety Level 2–3 laboratory.

However, if the species involved does not appear obvious, an epidemiological investigation should be performed alongside. The incubation period would have been too short in the present instance (<5–7 days) for the implication of *Rickettsia slovaca*, the second most frequently observed Rickettsial disease in France (14). Although it is endemic in north-eastern France, the clinical pattern provided by *R. slovaca* does not correspond to the one reported above. *R. slovaca* infection usually leads to the development of cervical or occipital lymphadenopathy with a single scalp eschar (formerly known as Tibola: tick borne lymphadenopathy) (Table 2).

*R. conorii*, the most frequently observed species in southern France, is also endemic in South Africa and in Eswatini and could, therefore, be implicated if the patient was infected while there (Fig. 1C). However, it did not correspond as closely as *R. africae* to the clinical pattern observed in our case study, associating two necrotic eschars without any secondary maculopapular rash (6). Thus, African tick-bite fever remained as the most plausible diagnosis in our case, especially if we consider that 80% of patients contract the disease after an international travel to South Africa (5, 15). The presence of clusters of cases among co-exposed persons could constitute a supplementary argument for the diagnosis (5) within the limitations of the application of mosquito repellent creams or sprays for clothing, such as here.

In order to establish a diagnosis of the *Rickettsia* species involved in our case, blood serological and PCR assays of the abdominal necrotic eschar were performed. The other microbiological diagnostic methods (7), that is, culture sometimes associated with matrix-assisted laser desorption/ionization time-of-flight assays performed on the eschar biopsy or blood samples (16, 17) and PCR assays directly on blood samples (18), were not performed here. Even if anti-*Rickettsia* spp. antibodies do appear in the patient’s blood (15), the available serological assays (commercially available chemiluminescent assays and, to a lesser extent, National Reference Centre indirect immunofluorescence assays) could not discriminate anti-*R. africae* antibodies from antibodies against other spotted group *Rickettsia* because of extensive cross-reactivity (5–7). Some authors have suggested using the cross-reactivity of commercially available CE serological kits, such as Virclia and Vircell, which we used (Table 1) for the diagnosis of African tick-bite fever (5), but it did not allow ensuring the implication of *R. africae* in cases where the species involved is discussed. The PCR assay performed on the eschar biopsy (or swab) is able to discriminate between species and confirm here the identification of *R. africae* (5–7). Interestingly, and even with early treatment with doxycycline, the serological window was a bit shorter in our case than the 3-week period classically reported for *R.africae* (5, 7).
